# The Prediction of Rare Clear Cell Carcinomas of the Liver via Pathological Component-Based Image Evaluation: A Case Report

**DOI:** 10.7759/cureus.91059

**Published:** 2025-08-26

**Authors:** Junya Ohnishi, Shoji Oura, Hiroshi Shintani

**Affiliations:** 1 Department of Surgery, Kishiwada Tokushukai Hospital, Kishiwada, JPN

**Keywords:** abundant cytoplasm, clear cell hepatocellular carcinoma, internal high echoes, retained rim enhancement, unchanged posterior echoes

## Abstract

A 79-year-old woman with a history of hepatitis C was referred to our hospital for detailed examination of her hepatic mass. Computed tomography of the hepatic mass showed very weak ring enhancement only on portal and late-phase images. Ultrasound revealed a well-circumscribed mass with unchanged posterior echoes, a presumed thin capsule, and predominantly iso- to high internal echoes, including focal areas with slightly lower background echoes. Magnetic resonance imaging of the hepatic mass showed low signals on fat-suppressed T1-weighted images, high signals on fat-suppressed T2-weighted images, and faint ring enhancement through early-to-late-phase images on dynamic studies. These image findings led us to speculate that the tumor had both edematous fibrous components in its center and tumor cells with abundant cytoplasm and some kind of intracellular substances, i.e., possible clear cell hepatocellular carcinoma. We, therefore, resected the hepatic mass laparoscopically. The pathological study showed that the mass had a lobulated shape, edematous fibrous components in its central areas, and atypical cells with clear cytoplasm proliferating in both trabecular and solid fashions. Immunostaining of the tumor revealed hepatocyte, arginase 1, CK20, CD10, CA9, and TFE3 negativity, along with AE1/AE3 and CK7 positivity, leading to the diagnosis of clear cell carcinoma of the liver. The patient recovered uneventfully and was discharged on the sixth postoperative day. Diagnostic physicians should note that pathological component-based image evaluation can predict histological characteristics even for very rare clear cell carcinomas of the liver.

## Introduction

Possible bleeding on a core needle biopsy [1] or a difficult approach due to mass location sometimes makes hepatocellular carcinomas (HCCs) be surgically treated without preoperative pathological diagnosis. Especially, small presumed HCCs are prone to being operated on both for diagnosis and treatment without a preoperative pathological diagnosis, which can unfortunately lead to over-treatment.

Computed tomography (CT) [2], magnetic resonance imaging (MRI) [3], and ultrasonography (US) [4] are the mainstays in the diagnosis of HCCs, similar to the image diagnosis for other solid malignancies. Positron emission tomography (PET), which provides metabolic information about target lesions, is widely used to make surgical indications for small lesions in various organs. However, similar to normal hepatic cells, the abundant phosphatases often present in small HCCs can provide physicians with incorrect information about the aggressiveness of HCCs on PET [5], frequently leading to a less frequent application of preoperative PET evaluation for such cases.

Diagnostic physicians generally make their image diagnosis through empirical pattern recognition, similar to that used for artificial intelligence diagnosis. Pattern-based image evaluation, however, may miss rare tumors; pathological component-based evaluation (e.g., fibrous vs. cellular components) can improve the detection of uncommon HCC subtypes. In short, understanding how pathological components, such as fat, mucus, and fibrous components, are depicted in each image modality may allow physicians to possibly diagnose atypical or rare cases [6].

Clear cell HCCs are a rare subtype that accounts for 3-5% of all HCCs [7]. Although clear cell HCCs have been evaluated pathologically [8], no imaging evaluations have been conducted on them due to their low incidence. Diagnostic physicians, therefore, rarely include clear cell HCCs in the differential diagnosis of liver tumors.

We report a rare clear cell HCC in which combined CT, MRI, and US evaluation predicted its histology via pathological component-based image evaluation.

## Case presentation

**Figure 1:**
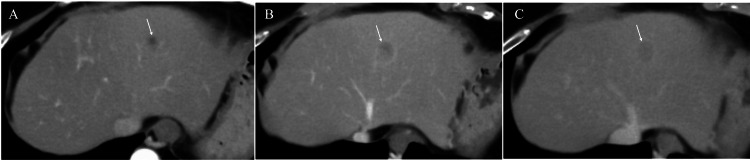
Computed tomography (CT) findings (A) CT showed no clear enhancement of the mass (arrow) on arterial phase images. (B) CT showed a very weak rim enhancement (arrow) on portal phase images. (C) CT showed retained nominal enhancement (arrow) on late-phase images.

A 79-year-old woman with a prior history of hepatitis C was referred to our hospital for detailed examination of her hepatic mass. Direct-acting antivirals had brought the patient a sustained virological response to her hepatitis C. However, periodic follow-up CT (slice thickness 5 mm) revealed a low-intensity mass, 1.5 cm in size, in the liver segment III, which showed very weak ring enhancement only on portal and late-phase images (Figure 1).

**Figure 2:**
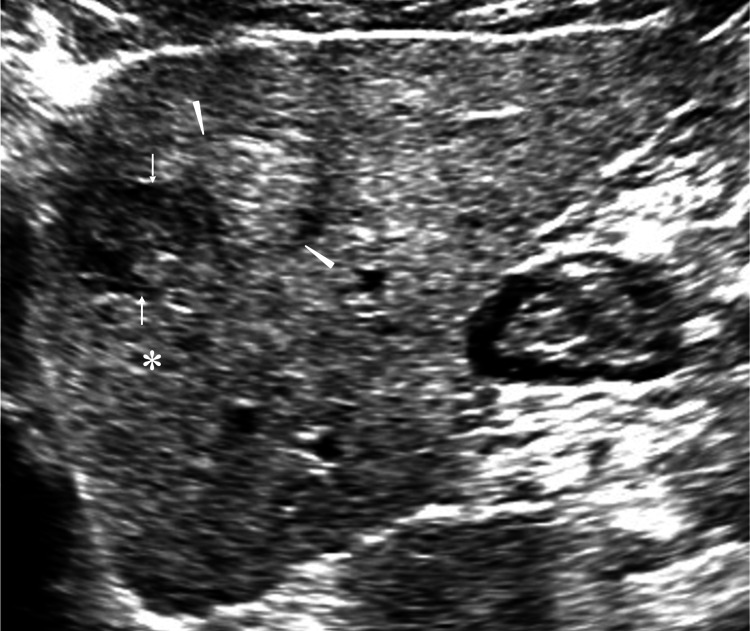
Ultrasound findings Ultrasound showed an oval mass (arrowheads) with predominantly internal high echoes and no enhanced/attenuated posterior echoes (asterisk). Slightly low internal areas (arrows) well corresponded to the edematous fibrous component areas (Figure 4B, asterisk).

Ultrasound revealed a well-circumscribed mass with unchanged posterior echoes, a presumed thin capsule, and predominantly iso- to high internal echoes, including focal areas with slightly lower background echoes (Figure 2).

**Figure 3:**
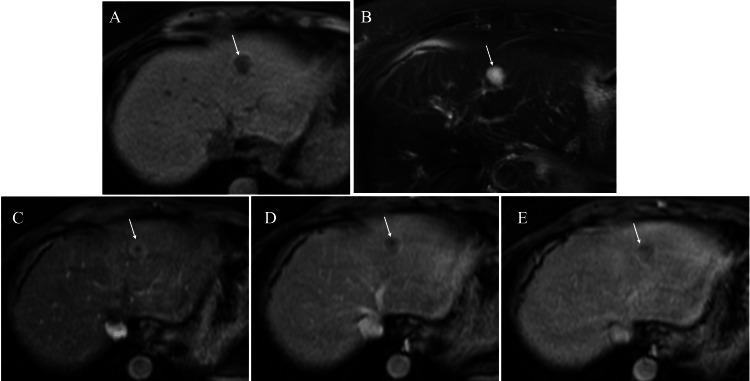
Magnetic resonance imaging (MRI) findings (A) Fat-suppressed T1-weighted images of the tumor showed low signals (arrow). (B) Fat-suppressed T2-weighted images of the tumor showed high signals (arrow). (C) Contrast images of the tumor showed rim enhancement (arrow) on arterial phase images. (D) Contrast images of the tumor showed very weak enhancement (arrow) on portal phase images. (E) Contrast images of the tumor showed retained very weak enhancement (arrow) on late-phase images.

MRI of the hepatic mass showed low signals on fat-suppressed T1-weighted images, high signals on fat-suppressed T2-weighted images, and faint ring enhancement through early-to-late-phase images on dynamic studies (Figure 3).

**Figure 4:**
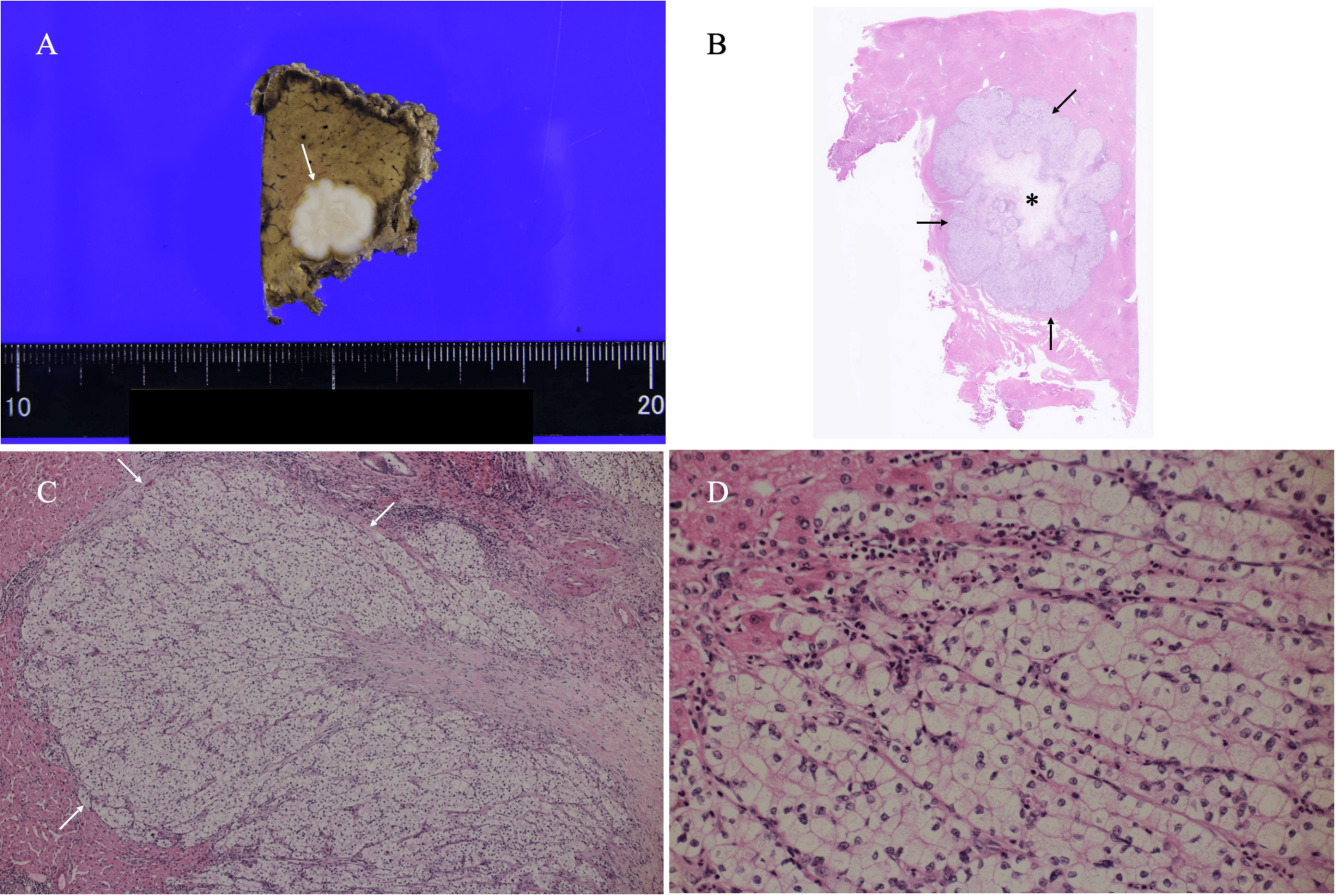
Pathological findings (A) The bisected cut surface of the tumor showed a micro-lobulated whitish mass (arrow). (B) A low-magnification view showed that the tumor (arrows) had a microlobulated shape and edematous, fibrous components in its center (asterisk). (C) A magnified view showed atypical cells growing in solid and trabecular fashions (arrows). (D) A further magnified view showed atypical cells with clear cytoplasm growing in a trabecular fashion.

Blood tests showed that α-fetoprotein (AFP) and PIVKA-II were within the normal range, but the lectin-reactive fraction of AFP was slightly elevated at 30% (Table 1). These image findings led us to speculate that the tumor had both edematous fibrous components in its center and tumor cells with abundant cytoplasm and some kind of intracellular substances, i.e., possible clear cell hepatocellular carcinoma. We, therefore, resected the hepatic mass laparoscopically. Pathological study showed that the mass had a lobulated shape, massive edematous fibrous components in its central areas, and atypical cells with clear cytoplasm proliferating in both trabecular and solid fashions (Figure 4).

**Table 1:** Blood tests AST: aspartate aminotransferase; ALT: alanine aminotransferase; LDH: lactate dehydrogenase; ALP: alkaline phosphatase; GTP: gamma-glutamyl transferase; Ch-E: cholinesterase; CK: creatine kinase; WBC: white blood cell; RBC: red blood cell; Hb: hemoglobin; Ht: hematocrit; CEA: carcinoembryonic antigen; CA19-9: carbohydrate antigen 19-9; AFP: alpha-fetoprotein; N.E.: not evaluated

Parameter	Reference Range	Before Operation	After Operation
Total bilirubin	0.4–1.5 ng/dL	0.8	1.0
Direct bilirubin	0.1–0.3 ng/dL	0.2	0.2
AST	13–30 U/L	35	34
ALT	7–23 U/L	39	30
LDH	124–222 U/L	208	198
ALP	38–113 U/L	45	49
Y-GTP	9–32 U/L	17	16
Ch-E	201–421 U/L	304	255
CK	41–153 U/L	254	101
Amylase	33–132 U/L	49	62
Total protein	6.6–8.1 g/dL	7.6	7.4
Albumin	4.1–5.1 U/dL	4.7	4.6
WBC	33–86 10^2^/µL	106	99
RBC	386–492 10^4^/µL	455	419
Hb	11.6–14.8 g/dL	14.2	13.5
Ht	35.1–44.4%	42.4	39.7
Platelet	15.8–34.8 10^4^/µL	26.8	25.2
CEA	0–5.0 ng/mL	2.7	N.E.
CA19-9	0–37 U/mL	19.1	N.E.
AFP	0–7.9 ng/mL	3	1.9
AFP isoforms			
L1	-	70	≥99.6
L3	0–10.0%	30	<0.5
PIVLA-II	0–28.4 ng/mL	15.8	13.4

**Figure 5:**
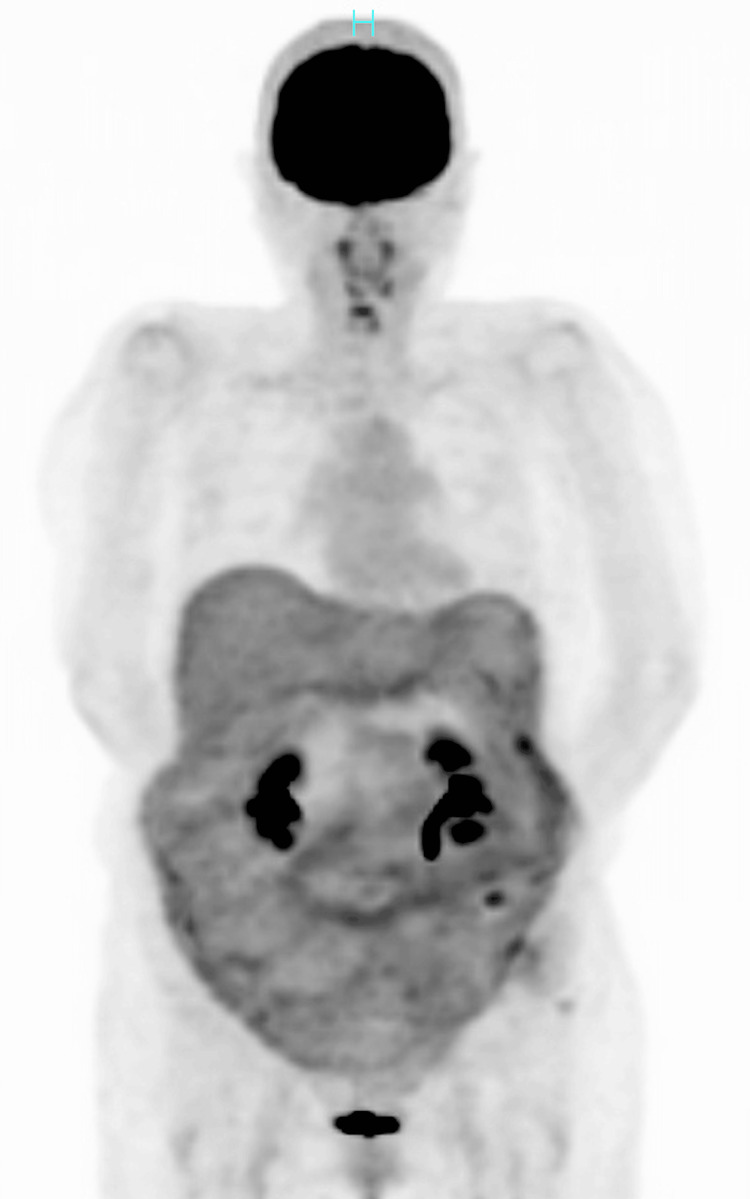
Positron emission tomography (PET) findings Postoperative PET showed no significant fluorodeoxyglucose uptake in the abdomen.

Immunostaining of the tumor showed hepatocyte, arginase 1, Melan-A, HMB-45, CK20, PAX8, CD10, CA9, and TFE3 negativity, as well as AE1/AE3 and CK7 positivity, leading to the diagnosis of clear cell carcinoma of the liver. The patient recovered uneventfully and was discharged on the sixth postoperative day. Postoperative PET/CT showed no fluorodeoxyglucose uptake in any organs, including the liver (Figure 5).

The patient is scheduled for follow-up on an outpatient basis over a period of five years.

## Discussion

It is well known that the tumor sizes of HCCs correlate well with their differential degree. In short, HCCs are more likely to be well, moderately, and poorly differentiated as the tumor size increases [9]. In addition, well-differentiated HCCs generally have portal vein-dominant blood flow and show delayed enhancement due to their slow delivery of contrast agents to the tumor. Well-differentiated HCCs, however, have sufficient enhancement at least in mid- and/or late-phase images, which markedly differs from the findings observed in this case.

Ultrasound wave backscattering generally requires a heterogeneous substance/cell with different acoustic impedance in contact or papillary/tubular structures [6]. Although the onset of enhancement varies from case to case, HCCs with papillary/tubular structures consistently demonstrate robust tumor enhancement on both CT and MRI. Internal high echoes, therefore, were not caused by the pathological papillary/tubular structures in this case. In our case, the focal high-echo areas on US corresponded to central edematous fibrosis on pathology (Figure 4B). We have already found that edematous, loose collagen fibers can generate high internal echoes [10], which correspond well to the punctate high signals against the focal low background areas in the fibrous component-rich areas, i.e., the mass center, on MRI in this case.

HCCs not otherwise specified generally have completely different images from those observed in this case, as mentioned above. Among the eight specific HCC subtypes [11], the steatohepatitic, clear cell, and chromophobe subtypes exhibit intra-tumoral or intracellular substances with markedly different acoustic impedance, either from HCC cells or from cells/substances surrounding cancer cells. Fat-suppressed T2-weighted MRI findings easily rule out the steatohepatitic subtype in this case. Both clear cell and chromophobe HCCs have light cytoplasm. We have already found that glycogen is one of the etiologies of bright cytoplasm formation in clear cell carcinomas [12]. We, therefore, can easily speculate that the cytoplasmic brightness in chromophobe HCCs is also generated by a similar mechanism. It, however, is well known that chromophobe HCCs have much less cytoplasm and, at least focally, more striking nuclear atypia than clear cell HCCs. Chromophobe HCCs, therefore, should have shown significantly more enhancement on both CT and MRI than clear cell HCCs, given their distinct differences from the image findings observed in this case.

Postoperative PET/CT to rule out a possible metastatic clear cell carcinoma in the liver further confirmed the diagnosis of primary clear cell HCC. It is well known that abundant phosphatase activities in well-differentiated HCCs make the FDG accumulation low due to the accelerated FDG excretion to the extracellular space through glucose transporters on the HCC cell membranes [5]. PET/CT, therefore, may help diagnose suspected HCC cases that show no enhancement in their central areas up to the late phase on CT/MRI, such as this case, which is characteristic of clear cell HCCs.

## Conclusions

Diagnostic physicians should note that rare clear cell HCCs have clear margins, peripheral weak rim enhancement only in portal/delayed images, both on CT and MRI, and internal iso- to high echoes with preserved posterior echoes. In addition, PET/CT may help diagnose suspected HCC cases as clear cell HCCs when these image findings are observed.
